# Construction of Knowledge Graph of 3D Clothing Design Resources Based on Multimodal Clustering Network

**DOI:** 10.1155/2022/1168012

**Published:** 2022-06-02

**Authors:** Jia Zheng, Wei Hong

**Affiliations:** ^1^School of New Media Art, Xi'an Polytechnic University, Xi'an, Shaanxi 710048, China; ^2^School of Information and Communications Engineering, Xi'an Jiaotong University, Xi'an, Shaanxi 710049, China

## Abstract

The construction of 3D design model is a hotspot of applied research in the fields of clothing functional design system teaching and display. The simple 3D clothing visualization postprocessing lacks interactive functions, which is a hot issue that needs to be solved urgently at present. Based on analyzing the existing clothing modeling technology, template technology, and fusion technology, and based on the multimodal clustering network theory, this paper proposes a 3D clothing design resource knowledge graph modeling method with multiple fusion of features and templates. The position of each joint point is converted into the coordinate system centered on the torso point in advance and normalized to avoid the problem that the relative position of the camera and the collector cannot be determined, and the shape of different collectors is different. The paper provides a multimodal clustering network intelligence method, illustrates the interoperability of users switching between different design networks in the seamless connection movement, and combines the hybrid intelligence algorithm with the fuzzy logic interpretation algorithm to solve the problems in the field of 3D clothing design service quality. During the simulation process, the research scheme builds a logical multimodal clustering network framework, which integrates compatibility access and global access partition fusion of style templates to achieve information extraction of clothing parts. The experimental results show that the realistic 3D clothing modeling can be achieved by layering the 3D clothing map, contour features, clothing size features, and color texture features with the modeling template. The developed ActiveX control is mounted on MSN, and the system is compatible. The performance and integration rate reached 77.1% and 89.7%, respectively, which effectively strengthened the practical role of the 3D clothing design system.

## 1. Introduction

With the development of e-commerce, online shopping has become an increasingly popular shopping method, and the online market of clothing products has also expanded. Ordinary products can be fully described only by text introduction and appearance pictures, while online sales of clothing products have their particularities [1–4]. First of all, in order to observe the clothing in a comprehensive three-dimensional manner, it is not only necessary to meet the customer's flat visual requirements for the product, but also the comprehensive three-dimensional display of the clothing product is particularly important; secondly, the customer needs to try on the clothing to see whether the clothing fits well and how the matching effect is; finally, it is also a particularity of clothing products to buy clothes together in order to consult each other [5].

However, there are still many defects in the current online virtual 3D clothing design room: first, the 3D clothing design system generally adopts the garment stitching technology, and the time complexity of the clothing modeling algorithm is relatively large, which affects the real-time performance of the system [6–9]. The system is located on the central server. In the face of a large number of clients, this one-to-many model can easily cause bottlenecks due to the flow of a large number of packets on the server node link, resulting in slow response speed and affecting the timeliness of the system. Purchasing clothes for mutual consultation is the particularity of clothing commodities. In real life, consumers watch the effect of 3D clothing design and communicate with each other, but the current network-based 3D clothing design system has not yet reflected this requirement [10].

Based on the 3D clothing resource template, according to the characteristics of different styles of clothing, this paper integrates the loose feature with the 3D clothing resource template to realize the geometric structure construction of the clothing modeling template and fuses the style features with the modeling template to realize the modeling template. The topological structure of the model is constructed; the size feature is integrated with the 3D clothing resource template, and different construction methods are used for different parts of the modeling template to realize the construction of clothing modeling templates with different styles and structures. By studying the general mode of virtual 3D clothing design room, making a detailed demand analysis of online 3D clothing design, and examining the shortcomings of existing implementations in application, this paper proposes a 3D clothing display system based on an instant messaging platform. The system uses three-dimensional clothing resource measurement technology, computer graphics, OpenGL, COM technology, instant messaging, and other technologies to dress the surface of the scanned three-dimensional clothing resource as a clothing model, which effectively overcomes the problem of large time complexity of clothing modeling algorithms. After three-dimensional clothing resource feature extraction, attitude synchronization, collision detection, and response processing, the realistic three-dimensional clothing shape simulation under lighting and material is finally realized. Using COM technology to develop 3D clothing design system controls is applied to instant messaging tools (MSN). Finally, through instant messaging tools, customers can observe the effect of trying on various styles of clothing from multiple angles and communicate with each other and achieve the purpose of collaborative shopping.

## 2. Related Work

The use of 3D virtual clothing technology in the clothing field can simulate the production process of sample farmers to shorten the design time of salary clothing, thereby greatly reducing the cycle of ready-made clothing. At the same time, the 3D clothing virtual technology provides a new sales channel for clothing sales—online shopping. Online clothing shopping is different from the traditional clothing sales model; it greatly reduces the cost of sales and provides convenience for customers. The three-dimensional clothing simulation technology makes it possible to launch new clothing online [11–13].

Liao et al. [14] believe that parametric design generally means that the structure and shape of the design object are relatively fixed, and a set of parameters can be used to define the size relationship. Modification of design results is driven by dimensions. The most commonly used serialized standard parts in production belong to this type. Gao et al. [15] found that feature modeling technology was nurtured and grown in the historical process that the development and application of CAD/CAM technology reached a certain level, and it was required to further improve the degree of integration and turnover of production organization. Yadav et al. [16] discussed that the eAD technology in the past can only describe the geometric information of the product, while the feature modeling is to better express the complete function and production management information of the product and serve for the establishment of the integrated information model of the product. Feng et al. [17] proposed to construct and edit clothing surfaces and curves based on the surface of the 3D clothing resource model to realize the personalized design of 3D clothing. The resource model is closely related and cannot be free-formed.

Phoha et al. [18] used a horizontal section to cut and intersect the entire 3D clothing resource surface at certain intervals and obtained a large number of iso-curves. The iso-lines were evenly segmented, and each subpoint was connected according to a certain topological relationship to obtain the clothing surface; then find out the key points of the 3D clothing outline position of the clothing from the segmentation points to form the feature control lines and realize the deformation of the surface through them [19]. On the surface of the 3D clothing resource model, the new surface obtained by moving the surface to the normal direction for a certain distance is used as the clothing surface, so as to design tight clothing, so as to realize free drawing of 3D curves on the surface of 3D clothing resources and then use these curves as boundaries to obtain clothing surfaces through interpolation [20–22]. This method is flexible and free, but due to the complex control of 2D to 3D conversion, it can only realize the design of simple clothing surfaces [23–26].

## 3. Resource Distribution of Multimodal Clustering Network

### 3.1. Network Description and Data Acquisition

The multimodal clustering network connects the vector data of other vertices and finds the vector whose cross-product with the horizontal vector is positive and has the smallest angle, and the corresponding vertex is used as the second vertex of the convex hull; then, connect the vectors of the remaining vertices. In order to realize the parametric modification of part of the size of the 3D clothing resource, the 3D clothing resource point data is layered, so that when modifying the 3D clothing resource model according to a certain parameter, it only needs to operate in the layer involved in the scope of the parameter, without having to go through each point in the model once.

(1) $$\begin{array}{c} \text{request}\left(i,j\right)=\left\{\text{image}\left(i,j\right),\text{image}\left(i+j-1\right),\text{image}\left(i+n-2\right),\ldots \text{image}\left(1\right)\right\}. \end{array}$$

In order to ensure that the estimated value of each parameter in the Gaussian mixture model is accurately calculated, when calculating the background parameters, all the hyper-multimodal element points in Tb and the background hyper-multimodal elements in Tu are selected as samples; in this way, according to the area selected by the foreground/background samples, K-Means clustering algorithm is used to model the foreground of the unknown area and perform the foreground modeling in other areas.

**Figure 1:**
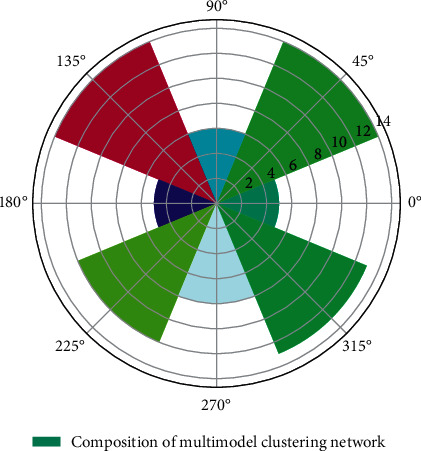
Data polarization distribution of multimodal clustering network.

In order to obtain an accurate 3D clothing map of clothing, and to solve the problem of accurate segmentation and real-time performance of the 3D clothing map, an improved graph cutting algorithm based on ultra-multimodal elements is used: first, a clustering algorithm is used to perform the 3D clothing map of Figure 1. After preprocessing, a super-multimodal element graph with uniform size and shape is obtained.

(2) $$\begin{array}{c} R_{n-i}^{i=0,1,2,\ldots ,n}\left(\frac{1-y\left(i,j\right)}{1+y\left(i,j\right)}\right)+R_{n+i}^{i-j=0,1,2,\ldots ,n}\left(\frac{1-f\left(i,j\right)}{1+f\left(i,j\right)}\right)=1. \end{array}$$

Each super-multimodal element corresponds to a node of the network graph, and the difference between adjacent pixels corresponds to the capacity on the edge, so as to construct a simplified network graph, replace the color value of the multimodal element points in the super-multimodal element with the RGB mean value in the super-multimodal element, and calculate the parameters of the Gaussian mixture model, thereby reducing the scale of the problem.

### 3.2. Feature Recognition of 3D Clothing Resource Model

Accurately represent quadratic regular surfaces with 3D clothing resources, so that regular and disc-shaped surfaces can be represented in a unified mathematical form, which cannot be achieved by other irrational methods. It has weights that can affect the shape of the curvilinear surface.

(3) $$\begin{array}{c} g\left(x,y\right)=\left\{\begin{array}{c} 1-y\left(i,j\right),\;y<x\\ R_{n-i}^{i=0,1,2,\ldots ,n}\left(\frac{1-y\left(i,j\right)}{1+y\left(i,j\right)}\right),\;y>x \end{array}\right.. \end{array}$$

Therefore, the shape is more suitable to be controlled and realized: the hierarchical structure enhances the influence of the local area on the classification results, and the clothing design resource sequence is divided into multiple subsequences to be learned separately and then combined.

**Figure 2:**
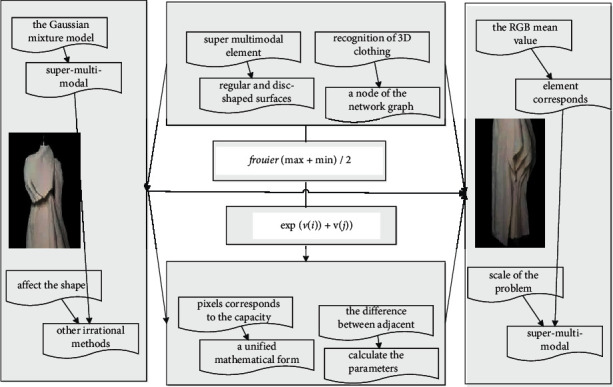
Feature framework of 3D clothing resource model.

The principle of texture mapping is to establish the mapping relationship between the texture pattern in the two-dimensional texture space and the object exposed point in the model space and map the texture pattern in the texture space to the surface of the two-dimensional or 3D object according to the algorithm in Figure 2. That is, the texture is pasted to the surface of the object through the coordinate transformation between the texture space and the object space.

(4) $$\begin{array}{c} g^{i-j}\left(x,y\right)-\frac{i!}{i\left(i-1\right)\left(j-1\right)!}=\frac{i!}{i\left(i-1\right)!/\left(j-1\right)!}. \end{array}$$

To complete this process, it is necessary to establish the corresponding relationship between the space coordinates (*x*, *y*, *z*) of the object and the space coordinates (*u*, *v*) of the texture, which can be described as *f*(*z*) by mathematical methods. Assume that the pattern is defined in one orthogonal coordinate system (*u*, *v*) in texture space, and the mapped model is defined in another coordinate system (*s*, 1). Generally, for the dimensional model space, (*s*, *t*) is the orthogonal coordinate system, and for the 3D model space, (*s*, 1) is the parameter coordinate space of the 3D orthogonal coordinate system; then, the actual mapping is the generation function.

### 3.3. Preprocessing of Clothing Data

After the 3D clothing map of clothing is segmented, the complete clothing area is obtained, and the color and texture features of the clothing are obtained at the same time. Using simple multimodal element binarization processing, the boundary between clothing area and nonclothing area can be extracted, and the clothing boundary in the form of multimodal element points can be obtained, and the three-dimensional clothing contour feature of shape can be obtained by using the 3D clothing contour tracking algorithm. After the 3D clothing map of clothing is segmented, the complete clothing area is obtained, and the color and texture features of the clothing are obtained at the same time.

(5) $$\begin{array}{c} p\left(u\left(i\right),v\left(i\right)\right)=\left[{\left(1-u\left(i\right)\right)}^{i-1}{\left(1-v\left(i\right)\right)}^{i-1}-{\left(1+u\left(i\right)\right)}^{i-1}{\left(1+v\left(i\right)\right)}^{i-1}-1\right]. \end{array}$$

**Figure 3:**
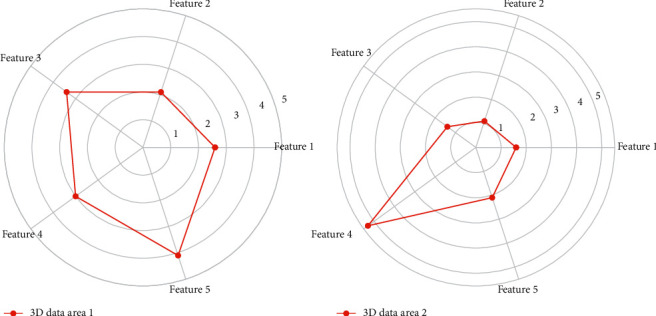
Radar map of 3D clothing data area division.

The more common method used in texture mapping is the screen scanning method, which is to visit the pixels in the order of the screen sweeping line and randomly sample the texture pattern. According to the segmentation results of the 3D clothing map, the seed points are determined interactively by means of dashes and dots, and in the image that has been divided into several regions after preprocessing, it is pointed out which regions are clothing and which regions are nonclothing. In this paper, the clothing area is marked with curves in the interaction process, and the areas in Figure 3 are marked with other curves.

The calculation process is as follows: first, calculate the point on the 3D curve that it represents according to the inverse perspective direction of the point to be displayed, and then according to the corresponding relationship between the plane 3D clothing map and the 3D surface, find out the point on the 3D curve on the 2D plane. The corresponding point in it obtains the grayscale or color of the point to be displayed from the vine. This process is actually a coordinate transformation process. The realization of the pattern texture effect on the surface of the garment is essentially the mapping of the fabric pattern on the surface of the object. Then, the features corresponding to multiple data types are cascaded to form a higher-dimensional feature vector containing information of various data types. Finally, the new feature is lost to the SVM model with stronger classification ability for training, and the recognition result is better than that of the Softmax model.

(6) $$\begin{array}{c} \sum_{i+j=c}^{i}{\left(-i\right)}^{i-1}{\left(1-u\left(i\right)\right)}^{i-1}+{\left(-i\right)}^{i-2}{\left(1-u\left(i\right)\right)}^{i-2}+{\left(-i\right)}^{i-3}{\left(1-u\left(i\right)\right)}^{i-3}=\frac{u\left(i\right)}{u\left(i-1\right)}. \end{array}$$

The skeleton method is used to represent the shape of the clothing, but it is not intuitive enough. At the same time, the details of the 3D clothing outline of some clothing cannot be accurately represented, which is not conducive to the extraction of clothing information. Using the 3D clothing contour representation can better represent the shape of the clothing in the 3D clothing Atlas, but in fact, the shape representation does not need to use all multimodal element points; as long as the 3D clothing outline is sampled uniformly, and *n* is sufficiently large, then it is enough to describe the original shape without losing detailed information. For some key points of shape characteristics, it can be added to the point set based on uniform sampling.

### 3.4. Multimodal Element Fitting Clustering

In multimodal elements, the *t* parameter specifies which texture should be defined, and this parameter is GLT. This function controls how wrapping is specified when texels are mapped to fragments, and how colors are filtered when texels in the texture do not correspond one-to-one with multimodal elements on the screen. The parameter *pe* describes the texture map format and data type, and the parameter pixels contains the texture 3D clothing map data, which is connected to the texture circle image itself and its boundaries.

(7) $$\begin{array}{c} q\left(i,j\right)=\sum p\left(i,j\right)\times \left(u\left(i\right)-v\left(i\right)\right)-p\left(i-1,j-1\right)\times u\left(i-1\right)\times v\left(i-1\right). \end{array}$$

The form of the blending function determines how the control points affect the shape of the curve, and the value of the parameter *u* ranges from 0 to 1. When *u*=0, the non-zero mixing function is B0, and 2 has the value 1. When *u*=1, the non-zero mixing function is B2, which has the value *l* at this point. In this way, the quadratic Bezier curve always passes through the control points VO and V2. The functions B1 and 2 affect the shape of the curve when the parameter *u* takes an intermediate value. Therefore, the generated curve is close to Vl. In this way, it is not possible for a Bezier curve to have local control over the shape of the curve. If you change the position of any one of the control points, the entire curve will be affected.

In the process of clothing shape matching in Table 1, after obtaining the shape three-dimensional clothing outline point set of the clothing three-dimensional clothing map and the style template by processing, generally take some points before and after the part shape feature point as the center as the shape feature segment. When matching, if the shapes near the feature points have similar curvatures and the same direction, and the Hausdorff distance between the point sets that make up the feature segment satisfies the given tolerance, then the two feature segments are similar in shape and can be matched.

(8) $$\begin{array}{c} \left[\begin{array}{ccc} \frac{p\left(i,j\right)}{q\left(i,j\right)} & \frac{p\left(1,j\right)}{q\left(1,j\right)} & \frac{p\left(i,1\right)}{q\left(i,1\right)} \end{array}\right]=p\left[i,j\right]. \end{array}$$

The multimodal element programming algorithm is used to solve the shape matching problem with the known order relationship of the point sets of the 3D clothing outline. Because the topological connection relationship information between the multimodal element point sets is fully utilized, the relationship between the local shape details can be better determined, so that more accurate matching results can be obtained. The disadvantage is that dynamic programming can only be used for shape matching between binary 3D clothing maps. It is divided into several subsequences in advance, including the action trajectories of multimodal elements at different times.

## 4. Construction of Knowledge Graph Model of 3D Clothing Design Resources Based on Multimodal Clustering Network

### 4.1. Node Distribution of Multimodal Clustering Network

Since the coordinates of the scanned data information and the system coordinates of the clothing design are inconsistent with the reference origin, and the coordinates of the 3D clothing resource model provide a positioning reference for clothing modeling, a unified coordinate system must be determined. This article adopts the following regulations. The horizontal direction *X* sets the left and right directions of the screen, and the vertical direction *Y* sets the up and down directions of the screen. According to the right-hand rule, the *Z* direction is set as the vertical screen outward. The XOY projection is the main view, the YOZ projection is the side view, the XOZ projection is the top view, and the other is the view space where the 3D clothing resource model can perform various transformations.

(9) $$\begin{array}{c} \text{imager}\left(r\left(i\right),r\left(j\right)\right)=r\left(i,j\right)-\frac{r\left(i\right)r\left(j\right)}{\sqrt{2}}+\frac{\text{rankeer}\left(r\left(i,j\right)-1\right)}{2}. \end{array}$$

The extraction of feature points of 3D clothing resource model is realized by a combination of automatic and manual methods. After the feature point information extraction is completed, the acquisition of the feature line is relatively simple. According to the position of the feature point, the intersection line of the surface and the plane of the 3D clothing resource can be obtained by cutting the surface through the section plane passing through the feature point, which is the feature line. The feature surface must be generated by fitting and reconstructing the data by using the information of feature points and feature lines.

(10) $$\begin{array}{c} C_{u\left(i,j\right)}^{v\left(i,j\right)}\left[r\left(i,j\right)-\frac{r\left(i\right)r\left(j\right)}{\sqrt{2}}\right]+C_{u\left(i,j-1\right)}^{v\left(i,j-1\right)}\left[r\left(i-1,j-1\right)-\frac{\sqrt{r\left(i\right)r\left(j\right)}}{\sqrt{1-i-j}}\right]+1=0. \end{array}$$

**Figure 4:**
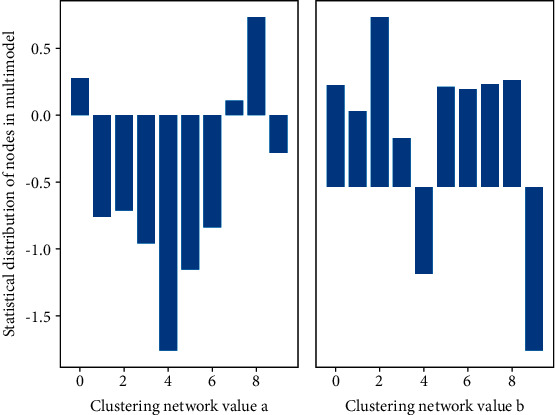
Statistical distribution of nodes in multimodal clustering network.

Each standard 3D clothing resource model has a corresponding size series, but the actual 3D clothing resource model is individualized and has no specific standards. It needs to be identified by feature points after data acquisition to calculate the feature size. The original scanned 3D clothing resource model is scanned by a 3D scanner and is usually accompanied by a feature information file as shown in Figure 4, including information such as artificially marked feature points. This auxiliary information brings some drawbacks to the subsequent processing modeling.

On the one hand, the characteristic information of artificial identification is inaccurate, and it needs to be reidentified and adjusted according to these data before it is used to process the 3D clothing resource model. On the other hand, it is extremely inconvenient to attach an information file to each 3D clothing resource model. Once lost or modified by mistake, the 3D clothing resource modeling process cannot be completed correctly. A more appropriate approach is to identify relevant features directly based on the 3D clothing resource data, rather than attaching other information. The remaining feature point sets are composed of corner points inside the image at different times. Therefore, it contains a lot of temporal information in the video sequence, and the HOG feature itself can describe the spatial information inside the image, so that the algorithm can also extract the spatiotemporal information of the video sequence feature.

### 4.2. 3D Garment Body Shape Information Design

When designing and cutting clothing, the “reference line” is the basis for cutting the garment. The so-called “reference line” is an indicator line marked on the three-dimensional clothing resource model, which is divided into two types: vertical and horizontal. In the top, the longitudinal reference lines are front and rear center line, side seam, and front and rear princess lines; horizontal reference lines are bust line, waistline, and hip line. These baselines divide the garment into pieces.

(11) $$\begin{array}{c} \left[\begin{array}{c} C_{u\left(i,j\right)}^{v\left(i,j\right)}\left(1-c\left(i\right)\right)\\ \ldots \\ C_{u\left(i,j\right)}^{v\left(i,j\right)}\left(i-c\left(i\right)\right) \end{array}\right]+\left[\begin{array}{c} \left(1-u\left(i\right)\right)\\ \ldots \\ \left(i-u\left(i\right)\right) \end{array}\right]=\left[\begin{array}{c} C_{u\left(i,j\right)}^{v\left(i,j\right)}\left(1-u\left(i\right)\right)\\ \ldots \\ C_{u\left(i,j\right)}^{v\left(i,j\right)}\left(i-u\left(i\right)\right) \end{array}\right]. \end{array}$$

**Figure 5:**
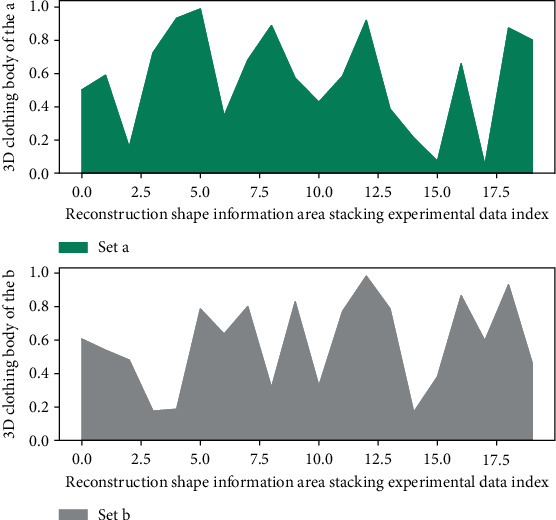
Area stacking of 3D clothing body shape information.

The front of the garment is divided into two symmetrical parts. In the right front half, the bust line, waistline, hip line, center line, and princess line divide the top into 6 parts. The left front half of the pattern can also be divided into 6 pieces corresponding to the right front half; that is, the front of the upper casserole is composed of 12 agricultural pieces as shown in Figure 5. The back of the garment is also composed of 12 symmetrical pieces.

Therefore, a top can be divided into 24 pieces. In trousers, the reference lines of trousers are waistline, hip line, side seam line, hole line, crotch line, and right (left) leg center line. The front of the trousers is divided into two symmetrical parts on the left. In the right front half, the reference line divides the trousers into 6 parts. Similarly, the left front half can also be divided into 6 pieces symmetrical to the right front half. The back of the trousers is also composed of 12 symmetrical agricultural pieces. Therefore, a trouser suit is divided into 24 parts.

(12) $$\begin{array}{c} A^{-1}\left(u\left(x\right),x\right)+B\left(u\left(x\right),v\left(u\right),x,y\left(x\right)\right)+C^{2}\left(v\left(x\right),y\left(u\right)\right)=1. \end{array}$$

After the learning mode is input into the neural network, the activation value of the 3D clothing resource point is obtained from the input layer through the multimodal element layer, and each 3D clothing resource point in the output layer is responded to. Then, according to the direction of reducing the error between the desired output and the actual output, from the output layer to the multimodal element layer and then to the input layer, the “error backpropagation” process is correcting the weights of each connection layer by layer: “forward propagation” process and “error backpropagation.” The network learning and training process is carried out at the intersection of the process; the global error of the network tends to be a minimum learning convergence process.

### 4.3. 3D Transformation of Resource Knowledge Graph

A collection of discrete sampling points on the surface of a 3D clothing resource model is acquired by a 3D scanner; we call it a point cloud. The system's three-dimensional configuration technology connects the point cloud data obtained by scanning into a triangular mesh model with topological structures such as points, edges, and surfaces, so as to describe the three-dimensional clothing resource model. In the research of this paper, the 3D scanning system is used to process the 3D clothing resources and the clothing on the surface of the 3D clothing resources and obtain the corresponding triangular mesh models of the 3D clothing resources and clothing models, respectively. The triangular mesh data of all models are saved as obi format file, and the 3D clothing display system reads the file and performs the data preprocessing process for the next application.

(13) $$\begin{array}{c} \langle \begin{array}{c} A^{-1}\left(u,x\right)+A^{-1}\left(u,x\right)=1\\ B^{-1}\left(v,y\right)+B^{-1}\left(v,y\right)=1. \end{array} \end{array}$$

**Figure 6:**
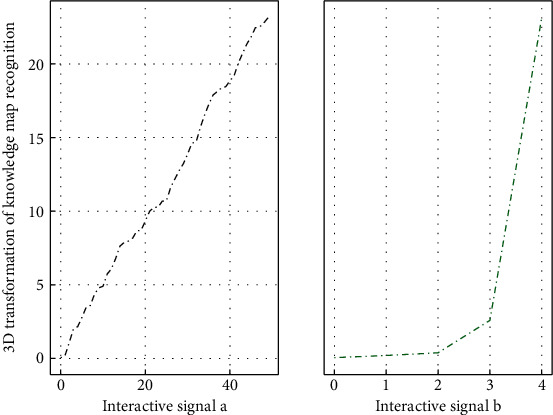
3D transformation of knowledge map of clothing design resources.

The data set is collected from the front by the Kinect camera and mainly includes three data types: RGB, depth, and bone. The sample data only describes the upper body information of the collected person. DEVISIGN contains a total of 24,000 clothing design resources, completed by 8 people (4 boys and 4 girls). Each clothing design resource contains only one instance of multimodal elements, of which four people collect twice for each dumb language action. Another four people collected once. The collectors maintain a standing posture at the beginning and the end, and the background of the collection is relatively clean, which makes the overall recognition difficulty in Figure 6 relatively low. The openGB provides two projection methods, projection and perspective projection, and, respectively, defines the corresponding functions and related parameters describing different projection methods.

Perspective projection is similar to observing things in daily life. The perspective effect makes objects farther away appear smaller. The line of sight (projection line) of perspective projection starts from the viewpoint (observation point), and the line of sight is not parallel. Perspective projection is divided into one-point perspective, beautiful point perspective, and three-point perspective according to the number of main vanishing points. Any bundle of parallel lines that are not parallel to the projection plane will converge into a point, called the vanishing point, and the vanishing point on the coordinate axis is called the main vanishing point. According to common sense, CAD software should choose orthogonal projection.

(14) $$\begin{array}{c} o\left(i,j\right)=\prod 2\left(u\left(i\right)-2\left(u\left(j-1\right)\right)+\text{iu}\left(j\right)\right)-\prod 2\sqrt{\left(u\left(j\right)-\text{iu}\left(j\right)\right)}. \end{array}$$

However, a sense of distance (depth in the *z*-axis direction) is indispensable in consideration of the effect of fitting a garment. Therefore, the system adopts perspective projection, which can adjust the depth direction of the garment in the virtual environment and enjoy the effect of fitting the garment from various angles. A special function is provided to realize the projection transformation.

## 5. Application and Analysis of Knowledge Graph Model of 3D Clothing Design Resources Based on Multimodal Clustering Network

### 5.1. Multimodal Clustering Network Texture Mapping

The wing edge data structure of multimodal clustering network is a data structure based on edge representation. It completely records the two adjacent faces, two vertices, and two adjacent edges on each side of each edge with pointers. It describes the topological relationship between points, edges, and faces of objects. For example, edge P1P2, its data structure contains pointers of 2 points, respectively, pointing to the 2 endpoints P1 and P2 of the edge, where P1 is the starting point of the edge, and P2 is its end point.

**Figure 7:**
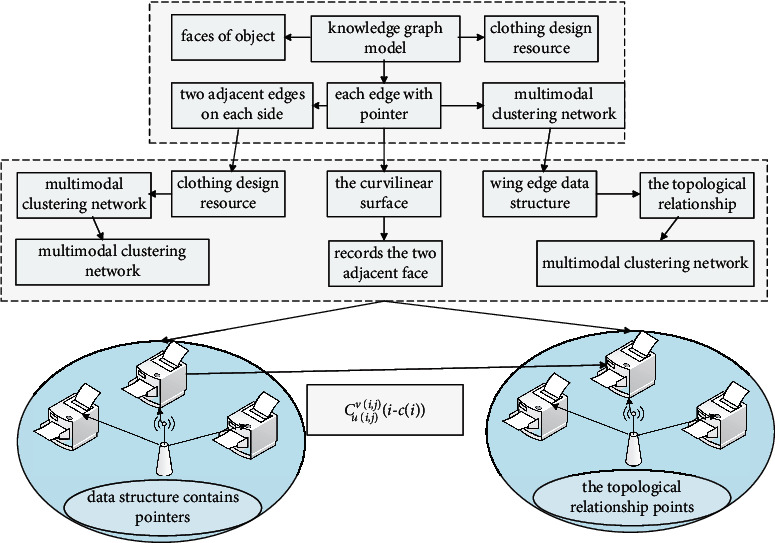
Node topology of multimodal clustering network.

In the regular shape shown in Figure 7, each edge is connected to two faces. Therefore, the data structure of the edge also contains two ring pointers, which, respectively, point to the outer edges on the two surfaces adjacent to the edge. In this way, the topological relationship between the shuttle edge and the adjacent surface is determined, in order to be able to start from this edge to find any other closed surface ring on which it is located. Edge: four edge pointers are also designed in the edge data structure, pointing to its “upper left edge,” “lower left edge,” “upper right edge,” and “lower right edge.”

(15) $$\begin{array}{c} \forall c,t\in R\left(c^{2}+t^{2}=1\right),\exists \max\left(c+t\right)\subset M\left(c,t\right). \end{array}$$

Obtaining the cross section ring from the connection relationship can ensure the correctness of the connection sequence of each vertex of the cross section ring, so the exact shape of the cross section ring can always be obtained. However, with this method, only the section ring closest to the given point can be searched in one cycle process; if there are multiple section rings, the calculation needs to be repeated many times. In addition, using the method of obtaining the section ring based on the connection relationship, only part of the section line can be obtained when the current section ring is not closed.

### 5.2. Realization of 3D Clothing Resource Knowledge Graph Simulation

Then, we use Gaussian kernels with different variances to perform different degrees of convolution processing on each color channel, so as to obtain the scale space under the three color channels and then calculate the normalized squared Laplace under the scale space for each scale. Points and ridges are obtained by normalizing the local maxima of the measure with ridge strength. Modify the style settings of the 3D clothing resource window, and set the bitmap format of the OpenGL drawing window and the subwindow to the same format as the corresponding RC; otherwise, it is easy to make mistakes. So, OpGL drawing windows need WS CREN and WS CLGS styles, formatting multimodal elements and creating rendering environments. To use OpL in a window, you also need to select an appropriate multimodal element format (Pixel Foat) for it. In the graphics operation steps of aIpenGL, the final link is to rasterize the graphics; that is, convert the shape into the multimodal element information displayed on the computer screen, and write this information into the frame buffer to realize the screen display.

(16) $$\begin{array}{c} \overset{c,t<1}{\overset{︷}{\max\left(c+t\right),\max\left(c-t\right),\min\left(c+t\right),\min\left(c-t\right)}}⟶\frac{\text{frouier}\left(\max+\min\right)}{2}. \end{array}$$

**Figure 8:**
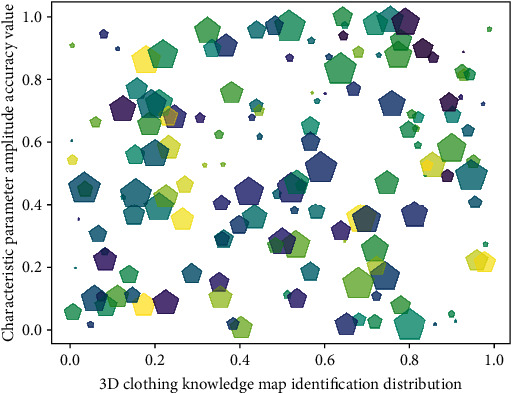
Recognition distribution of 3D clothing resource knowledge map.

From the above four similarities, it can be concluded that the background environment of the current mainstream multimodal element datasets is relatively pure, and there is no interference from other factors, thereby reducing the difficulty of identification caused by the acquisition conditions and prompting the algorithm to focus on the action of the multimodal element itself. In addition, the dataset in Figure 8 contains a variety of data types, which is convenient for the algorithm to capture the action characteristics of multimodal elements from multiple angles and improve the recognition rate of the algorithm.

Using function approximation modeling methods, a suitable function must be assumed, which is difficult to do with complex multivariate systems. Neural networks are considered as universal systems that do not need to assume any patterns and can solve a wide range of problems with a general structure. In fact, some assumptions have been implicitly made for the problem to be solved in the neural network design.

(17) $$\begin{array}{c} \frac{\partial o\left(x\right)}{\partial y\left(x\right)}-\frac{d\left(x^{2}-2x-1\right)\left(x^{2}+2x+1\right)}{\partial x}=1. \end{array}$$

For example, linear problems use linear activation functions, and determining the number of layers of multimodal element layers and the number of 3D clothing resource points is also problem-dependent. The determination of the network connection weight requires a training data set and a large number of operations. The data set must reach a certain scale, and the correct distribution can represent the entire problem domain. Otherwise, obvious errors may occur when the trained network is used to process the source data. The method selects the angle information of each joint point of the skeleton to describe the posture information of the collected person at the current moment, so as to solve the problem of gesture action recognition in the dance process.

### 5.3. Example Application and Analysis

The multimodal element classification layer depth map is divided into blocks of size 80×80, and each block contains four cells of size 40×40. Then, the gradient directions of all multimodal element points in each cell are calculated to obtain the gradient histogram corresponding to the cell. The histogram is divided into 16 intervals according to the gradient size. After normalization, each cell can get a 16-dimensional feature descriptor. The feature vector of the block can be obtained by concatenating the features belonging to the same block, and then concatenating the feature vectors corresponding to all blocks in Table 2 to obtain the final feature descriptor, that is, the HOG feature.

Compared with skirts, tops are more complicated to be divided into pieces. A more traditional method in clothing design is called the six-piece method; that is, the front and rear pieces of the top are divided into six pieces for cutting, and the other side is symmetrical with respect to the princess line; this method is extremely suitable for the design of sleeveless soil dress. After investigation, this method is not suitable for this paper, because some of the shards have five edges, which cannot be implemented using quadratic Bezier blue surfaces. However, this paper draws on this method and improves it, dividing the five-sided tunnel surface into two Bezier surfaces, so that it can be realized by Bezier surfaces.

(18) $$\begin{array}{c} m\left(i,j\right)={\left|i\times j-i-j-1\right|}^{-1}\times \;\exp\left(v\left(i\right)+v\left(j\right)\right),i,j=1,2,3\ldots ,n. \end{array}$$

This process still adopts the idea of histogram statistics. The RGB three-dimensional clothing map is converted into a grayscale image in advance and then divided into grids with a size of 6 × 8. The range of 0°∼180° is also divided into 16 intervals, and according to the direction of the optical flow vector, the number of multimodal element points that should be assigned to each interval is counted, and then the normalized multiple feature vectors are connected in series to form HOF features to describe the time information between two adjacent frames of 3D clothing maps.

**Figure 9:**
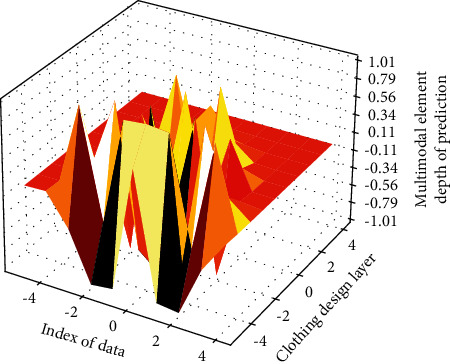
Depth distribution of multimodal element clothing design layer.

The model maps all the corresponding feature points in a picture to several preset types, thus forming a histogram vector of uniform dimension as the final feature. First, a hierarchical strategy is adopted for the object, and the clothing design resources are divided into several subsequences. Each subsequence in Figure 9 is used as a complete input unit, which is convenient for the model to capture more local information of the clothing design resources. Secondly, the input of the model is normalized, and the uniform random sampling method based on optical flow is combined with the input module with a hierarchical structure in the C3D network. Then, the multimodal data fusion strategy is adopted, and the three data streams of RGB, depth, and optical flow are, respectively, passed to the model, and the model learns the characteristics of various types of data and then fuses various data results in order to achieve better results. Finally, the pretrained C3D model is used to extract the output of the middle layer of the network as features, and the fused features are input to the model with stronger classification ability to realize the action recognition of 3D clothing resources.

## 6. Conclusion

In this paper, a multimodal element point 3D clothing design coordinate transformation model is established. The user provides their body shape information, and the model obtains the coordinates of the characteristic points of the clothing according to this information and obtains the curve control points of the clothing model that the customer wants to build based on the knowledge of air analysis geometry and the control point solving algorithm. First, the simulation scene and simulation result data are saved in the database, and the user can configure the main simulation scene data. Then, the corresponding actual simulation result data is determined accordingly, and the real-time clothing design function display is carried out. The extraction of feature points of 3D clothing resource model is realized by a combination of automatic and manual methods. Then, according to the biquadratic Bezier surface generation method, the line is formed from the point, and the surface is formed by the line, and the clothing model is generated. In order to allow customers to better experience the patterns and patterns of clothing and enhance the reality of three-dimensional clothing, texture mapping technology is used. At the same time, in order to allow temporal customers to appreciate the model from multiple angles, the paper also provides the design of coordinate transformation. The feature surface must be generated by fitting and reconstructing the data by using the information of feature points and feature lines. After the feature point information extraction is completed, the acquisition of the feature line is relatively simple. According to the position of the feature point, the intersection line of the surface and the plane of the 3D clothing resource can be obtained by cutting the surface through the section plane passing through the feature point, which is the feature line. However, the algorithm currently lacks the step of instance segmentation for the input video, which makes the algorithm unsuitable for continuous video sequences containing multiple gesture instances. Therefore, adding a fast and accurate gesture instance segmentation method to the preprocessing part of the algorithm is the next step and research focus.

## Figures and Tables

**Table 1:** Multimodal element fitting clustering description.

Element case	Clustering ratio
A1	0.219
A2	0.661
A3	0.318
A4	0.006
A5	0.807
B1	0.484
B2	0.743
B3	0.022
B4	0.594
B5	0.375
C1	0.086
C2	0.525
C3	0.437
C4	0.666
C5	0.117

**Table 2:** Multimodal clustering network algorithm steps.

Clustering network algorithm text	Feature descriptor description
Import matplotlib.pyplot as plt	*c*(*i*) to be divided into pieces.
From mpl_toolkits.mplot3d.axes3d import data	A more traditional method *i* − *u*(*i*)
From matplotlib import cm	Compared with skirts
Import numpy as np	*A* ^−1^(*u*, *x*) is called the six-piece method
Private final int freame_*x* = 50;	*y*(*x*) in clothing design
Private final int freame_*y* = 50;	The multimodal element
Private final int freame_width = 600;	max(*c*+*t*) is divided into
Plt.plot(time,b,“-”, label = “conductor 1”,linewidth = 2)	(*u*, *x*) blocks of size
Plt.plot(time,b1,“-”, label = “conductor 2”,linewidth = 2)	Depth map *u*(*i* − 1)
Plt.xlabel(“time(ms)”, fontsize = 14)	Classification layer

## Data Availability

The data used to support the findings of this study are available from the corresponding author upon request.
